# Time-Processing, Daily Time Management, and Autonomy in School-Age Children with ADHD Compared to Typically Developing Children and Children with Intellectual Disabilities—Different Patterns

**DOI:** 10.3390/children13010143

**Published:** 2026-01-20

**Authors:** Birgitta Wennberg, Anette Kjellberg, Per A. Gustafsson, Lena Almqvist, Gunnel Janeslätt

**Affiliations:** 1 Child and Adolescent Psychiatry, Center for Social and Affective Neuroscience, Department of Biomedical and Clinical Sciences, Linköping University, 581 83 Linköping, Sweden; 2 Independent Researcher, 581 83 Linköping, Sweden; 3 School of Health, Care, and Social Welfare, Mälardalen University, 721 23 Västerås, Sweden; lena.almqvist@mdu.se; 4 Department of Public Health and Caring Sciences, Uppsala University, 751 22 Uppsala, Sweden; gunnel.janeslatt@regiondalarna.se; 5 Centre for Clinical Research Dalarna, Uppsala University, 791 31 Falun, Sweden

**Keywords:** ADHD, adolescent, autonomy, child, cluster analysis, intellectual disability, occupational therapy, participation, time management

## Abstract

**Highlights:**

**What are the main findings?**

- Children with ADHD, as a group, have lower time-processing ability and autonomy compared to typically developing children, but daily time management is also lower than that of children with ID.
- Children with ADHD and children with ID have the same overall pattern of time-processing ability, but it may be delayed in relation to the typically developing children.

**What are the implications of the main findings?**

- The delayed time-processing ability in children with ADHD could affect their daily time management and autonomy.
- The overall findings indicate the need to measure children’s time-processing ability and their daily time management to tailor a suitable intervention for each child who needs it.

**Abstract:**

Background: Children with ADHD and children with intellectual disability (ID) often have problems with daily time management (DTM). It is, however, less well-known how the underlying time-processing ability (TPA) may impact children’s DTM and autonomy. The purpose of this study was to investigate DTM, TPA, and self-rated autonomy in the activities of everyday life among children aged 9–15 years with and without disabilities. Methods: The participants were matched samples of children with ADHD (*n* = 47), with ID (*n* = 47), and typically developing (TD) children (*n* = 47). A descriptive, comparative, and cross-sectional design was used. Group comparisons with one-way analysis of variance (ANOVA), Tukey post-hoc tests, bootstrapping, and a cluster analysis were used to analyze the data. Results: Children with ADHD and children with ID had significantly lower TPA and DTM than TD children. Children with ADHD had even lower DTM than those with ID. Children with ADHD and ID have the same overall pattern of TPA, but it may be delayed, affecting their DTM and autonomy. However, there was considerable heterogeneity among the children with ADHD and ID, ranging from skilled to having significant problems in TPA. For all children, the levels of self-rated autonomy seemed to follow the level of TPA. Conclusions: Children with ADHD and children with ID have an increased risk of delayed TPA, affecting their DTM and autonomy, which may also influence their participation in daily activities. The results indicate a need to measure TPA and DTM to tailor interventions for each child.

## 1. Introduction

### 1.1. Daily Time Management and Time-Processing Ability

Managing one’s time is important to perform everyday activities and to become autonomous and independent [1,2,3]. In the International Classification of Functioning, Disability and Health (ICF), managing one’s time could be found in the code carrying out daily routine (d230) in the Activity and Participation component [4]. According to the ICF, managing one’s time is primarily based on a person’s cognitive functioning related to time [2,4,5,6]. How a person manages time in everyday activities can also be influenced by environmental factors such as support from parents and school staff, as well as access to time-assistive products [4]. In the ICF, cognitive functions related to time could be found in the component Body functions and in the codes experience of time (b1802), orientation to time (b1140), and time management (b1642), included in higher-level cognitive functions (b164). A model of time-processing ability (TPA) and daily time management (DTM) has been developed to clarify how different cognitive functions related to time are linked to DTM [2,6,7,8]. The relevance of a broad, integrative, and functional model that could account for the cognitive process of time is mentioned in Accogli et al. [9]. The authors also state that the TPA model contributes to understanding time-processing (ibid). TPA develops during childhood and adolescence and seems to follow the developmental stages, as indicated by chronological age in children without disabilities [3,8]. TPA is a unidimensional construct containing three categories, which can be seen as hierarchical levels of complexity [7]: time perception is the basic level, followed by time orientation (divided into two sub-categories: time concepts and objective time), and the top level of TPA, time management [7]. Time perception is, in this model, expanded to the subjective experience of durations of activities, and time orientation includes telling time and knowing what day and month it is [2,6]. Time management, defined as ordering events in chronological sequences and allocating the appropriate amount of time for activities, is an important part of executive functions [4,6]. Executive function refers to brain functions that activate, organize, integrate, and manage other functions [10,11]. It enables individuals to account for short- and long-term consequences of their actions and to plan for the subsequent results. It also allows individuals to make real-time evaluations of their actions and make necessary adjustments if those actions are not achieving the desired result. The concept of executive functioning could be regarded as an underlying capacity for autonomy and for the sub-concept of time management within the cognitive function of TPA. Transferring the cognitive functions into managing one’s time in daily life (DTM) is a challenge and takes a lot of practice.

In a study of young children aged 5–10 years, with and without disabilities, the results showed four typical patterns related to the chronological age of the children without disabilities [8]. Children with and without disabilities were present in all cluster patterns, but children with a diagnosis were significantly older in all clusters except for the group with the most skilled children. Parents’ rating of their child’s DTM was related to the child’s TPA. The more skilled the child was in TPA, the higher the parent rated the child’s DTM (Ibid).

### 1.2. Cognitive Impairment, Autonomy, and Participation

Cognitive impairment affecting TPA is not always visible but can still lead to reduced autonomy and participation in everyday life. According to Cardol, De Jong, and Ward [12], personal autonomy can have two dimensions: deciding what to do (decisional autonomy) and doing what one has decided (executional autonomy). Decisional autonomy loads heavily on cognitive functions, i.e., to know one’s capacity and preferences, to know the demands of the activity, to take into account pros and cons, and finally to decide what to do and when to do it [13]. Executional autonomy, according to Peny-Dahlstrand et al. [14], is likewise dependent on the ability to initiate, organise, adapt, and proceed with the task until the goal is reached, i.e., observable executive functions. In their study of children with spina bifida (6–14 years old), children’s levels of autonomy were strongly related to the performance of everyday activities and to activities depending on executive functions. Rosenberg [15] also states that executive functions are vital for young children’s independence and autonomy in daily life. Also, in Janeslätt et al. [8], the children’s self-rating of autonomy was related to their TPA, except for the youngest children (5 years).

Autonomy is suggested to be a prerequisite for an individual’s ability to participate in his or her current environment [12,16], and high self-ratings of autonomy have been shown to be related to a high degree of self-rated participation [17,18] and good quality of life [19]. Personal characteristics such as autonomy and internal locus of control have been related to participation at school for children with disabilities [17].

### 1.3. Children with ADHD and Difficulties with Time

Several researchers have emphasised deficiencies in the executive functions as a basic problem for children with ADHD [10,20]. Children with ADHD have been shown to have a delayed maturation of the cerebral prefrontal regions, which are important for executive functions, including time management [21,22]. Children with ADHD also seem to have a different sense of time than typically developing children [23,24]. Their ability to discriminate and reproduce time intervals and to make retrospective time estimations has been shown to be impaired [25,26,27]. The perception of time is mainly researched in experimental studies [26,28,29]. Mioni et al. [30] found that children with ADHD performed less precisely in time-based prospective memory tasks and that time perception predicted prospective memory accuracy. Children with ADHD may also have reduced ability to value time in relation to the actual duration of daily activities [8]. These difficulties are apparent mainly in the experience of time, and planning and organising the plan when carrying out daily activities, at home, at school, and during play [31]. In a study of Lindblad and colleagues’ [32] children with ADHD, 6–16 years, had lower adaptive functioning compared to children with mild intellectual disabilities (ID). Children with ADHD also seem to have difficulties in the representation and correct use of time concepts. In a study of De la Charie and colleagues [33], where 33 children with ADHD aged 8–12 were matched in age and gender with children without disabilities, it was found that children with ADHD had significantly lower Time knowledge. In an RCT study involving 38 children aged 9–15 with ADHD, a significant increase in both TPA and DTM was observed after training in TPA and compensation with time-assistive products [34]. In another study, children of the same age also achieved their time-related activity goals after the same intervention [35].

### 1.4. Children with ID and Difficulties with Time

Children with ID have cognitive development, including the development of TPA, that is slower than in children without disabilities, and the level of abstract thinking as an adult is lower [36,37]. It is well known that difficulties with abstract concepts, such as the understanding of time concepts [36,38] and a reduced working memory [36], are common in children and adults with ID. This affects the practical skills in activities in daily life [37]. Clinically, children with ID have been targeted with interventions directed towards time deficits for many years, but nevertheless, there are few studies on TPA and DTM [38]. In one study involving children aged 5–10 years with different diagnosis, children with ID (*n* = 20) did show lower TPA than TD children and children with other diagnosis [8] and a study by Sköld and Janeslätt [39] children with mild ID age 10–17 (*n* = 19) did have a lower TPA than children with cerebral palsy, autism, ADHD and a lower self-rated DTM than children with cerebral palsy. Also, in one of the few studies involving children with ID, the children made more errors in prospective memory tasks than children without disabilities [40]. They also had a weaker time reproduction than children without disabilities. It has been shown that children and adults with ID benefit from time-assistive products to enhance DTM and TPA [41,42,43] and that children with ID aged 10–17 years can improve their TPA by training in low levels of TPA that focus on time perception and the duration of activities [44].

### 1.5. Self-Rated Autonomy in Children with ADHD and Children with ID

Autonomy in children with disabilities is mostly studied in a school context. Results show lower participation and self-rated autonomy in children with disabilities compared to children without disabilities [18,45]. In Eriksson et al. [45], 66 children aged 7–12 years with and without disabilities were observed at school for one day and were also interviewed about participation and asked to self-rate their autonomy. The results showed that children with disabilities, mostly children with ID, ADHD, and children with motor impairment, had lower participation and self-rated autonomy compared to children without disabilities. In Eriksson and Granlund [18], a comparison between 959 children with and without disabilities aged 7 to 17 years, children with disabilities, including children with ID, rated their participation and autonomy in school activities lower than children without disabilities. Studies in children with ADHD and children with ID self-rating their autonomy outside the school context are sparse. We only found two studies. In Janeslätt et al. [2], 118 children with different diagnoses, including ADHD and ID, aged 6–11 years, self-rated their autonomy. Also, parent ratings of DTM and measurements of TPA were conducted. Results showed a significant relation between self-ratings of autonomy, parents’ ratings of DTM, and the children’s TPA. The same pattern was shown in Janeslätt et al. [8], where children with (*n* = 77) and without (*n* = 89) a diagnosis, aged 5–10 years, self-rated their autonomy. As in the previous study, autonomy and DTM were related to the children’s TPA. Children with disabilities were found in different clusters, showing diversity in TPA, DTM, and autonomy not explained by diagnosis.

Summing up the knowledge presented so far, there is convincing evidence from various researchers supporting that children with ADHD have difficulties with time in terms of time sense, the experience of time, and, according to the authors, related problems in carrying out daily activities compared to TD children. Also, in children with ID, difficulties with time as understanding of time concepts were reported, as well as improvement in DTM following time-related interventions. Still, no study was found that captured the variation in time-related function and autonomy across a broad developmental spectrum in children aged 9–15 with and without disabilities.

The aim of this study is to investigate TPA, DTM, and self-rated autonomy in everyday activities in children aged 9–15 years with ADHD, children with ID, and TD children.

Research questions:

1. Are there any differences, and if so, what, in TPA, DTM, and self-rated autonomy in everyday activities between children with ADHD, children with ID, and TD children?
2. Can different patterns of levels of TPA be identified in children with ADHD, children with ID, and TD children?
3. Do children within the same diagnostic category or within the same age span share membership of a group with a specific pattern of TPA?
4. Do the parent-rated DTM and the self-rated autonomy of children differ between patterns?

## 2. Materials and Methods

A descriptive, comparative, and cross-sectional design [46], with samples matched in age and gender, was used.

### 2.1. Participants

#### 2.1.1. Children with ADHD

The data from children with ADHD were collected for a randomized study [34] using a consecutive sampling when recruiting children with ADHD from three child and adolescent psychiatric clinics (CAPs) and one child habilitation service (HAB) in Sweden between September 2012 and March 2015. In the current study, baseline data from both the intervention and control groups were used for all children. Inclusion criteria were a diagnosis of ADHD determined in accordance with DSM-IV criteria by an experienced CAP specialist after a thorough neuropsychological investigation encompassing careful clinical examination and questionnaires, a steady prescription of ADHD medication for at least three months, age 9–15 years and parent-reported difficulties with DTM, and ten points or more on a clinical rating of 15 statements related to problems with DTM (a subscale of the Five to Fifteen parent questionnaire [47]. Examples of statements were if the child was stressed by time limits, had difficulties in using a watch, or if the child was “lost in his/her own world” and was not aware of the passage of time. Further examples were when a child had obvious difficulties in carrying out and completing morning routines and arriving in time for school, or had difficulties in calculating the time span for daily activities and leisure activities. Exclusion criteria were ID (IQ < 70), autism spectrum disorder, or language barriers (e.g., not being able to answer questionnaires in Swedish).

This resulted in 47 children (mean age 11.5) with ADHD (17 female/30 male), of whom three had access to time-assistive products. Most of the children lived with both biological parents (53%), 15% lived in shared custody arrangements, 13% with one biological parent, 9% lived with one biological parent and a stepparent, and 10% other or no answer. A majority of the children’s parents were married or cohabiting (55%), 34% were divorced, 4% were widows, and 7% did not answer. The majority of the mothers (61%) and fathers (45%) had a university/college or high school/upper secondary school education, while 30% of the mothers and 41% of the fathers had vocational education or elementary school. Seven children had at least one parent who was born abroad.

Parents of the children were asked to participate in the study during ordinary monitoring visits to CAP or HAB. Those who agreed to participate were given written invitations to a personal visit for the data collection. Data collection was conducted with the child and the parent separately.

#### 2.1.2. Children with ID and TD Children

To capture variability in time-related functioning and autonomy across a broad developmental spectrum, both participants with intellectual disability and typically developing children were included in the study. To enable comparisons, each child with ADHD was matched, pairwise, with one child with ID (*n* = 47) and one TD child (*n* = 47) based on gender and chronological age. Children in the comparison groups were samples drawn from a database holding data collected between 2006 and 2015, containing data from children with different diagnoses (*n* = 200) and from TD children (*n* = 500). Data was gathered by certified occupational therapists working within Habilitation services and participating in courses for KaTid (see below) certification. The data from the comparison group of ID children were all from children within the ordinary work area of the occupational therapists.

The matching was performed based on the child with ADHD, who was matched in age and gender with one TD child and one child with ID. Only children with a complete KaTid assessment were chosen. If more than one child fit the child with ADHD in terms of gender and age, the child with data from both the Time-Parent scale and Autonomy scale was chosen; some of the children with ID lacked data from the Autonomy scale. All children with ID were diagnosed in accordance with DSM-IV criteria, having mild or moderate ID, and three of them had comorbidities (one child with autism spectrum disorder, one with cerebral palsy, and one child with hearing and language impairment). None of the children with ID were diagnosed with ADHD. Twenty-three of the children with ID had access to time-assistive products. TD children were recruited by trained and certified KaTid-raters from the normal population as a convenience sample, self-reported by the parents as being healthy, having no diagnosis, and with informed consent, and thereafter included in the database. None of these children was reported to have access to time-assistive products. In total, there were 141 participants.

### 2.2. Procedure

The children’s TPA was measured using a structured assessment (KaTid), and the children’s DTM was rated using a parent questionnaire (Time-Parent scale). The children’s autonomy was rated by self-reporting (the self-report Autonomy scale). Children’s TPA was assessed by a certified and clinically experienced occupational therapist in a meeting. The occupational therapist selected the KaTid version that suited the child’s age. For the TD children, KaTid-youth was used regardless of age. On the same occasion, all children in the sample filled in the self-assessment instrument (Autonomy). If needed, the occupational therapist made some adjustments in the self-assessment instrument by reading or explaining the statements. Also, if needed, the response alternatives were cognitively adapted using pictures. During the assessment occasion, the children did not have access to any time-assistive products. If the child requested it or if the occupational therapist deemed it necessary, a break was taken. For most children, the complete assessment was carried out using all instruments on one occasion, lasting for about 1 to 1 ½ hours, with a break when the child needed it. Only in exceptional cases was the assessment performed on two occasions, predominantly for children with moderate ID. In the meantime, the parents of the children in this study were asked to complete a structured questionnaire (Time-Parent scale) in which they assessed their children’s DTM.

### 2.3. Instruments

Kit for assessing time-processing ability (KaTid^®^), www.katid.se, Falun, Sweden [6] is an instrument for the assessment of time perception, time orientation (divided into the subcategories time concepts and objective time), and time management for children aged up to 10 years (KaTid-Child version) and adolescents aged from 10 years (KaTid-Youth version). It is a table-top test to be performed by a trained certified professional, e.g., an occupational therapist. The child responds to item questions and performs practical exercises. KaTid-Child contains 57 items measuring: time perception (15 items), time orientation (32 items), and time management (10 items), summarised into one measure of TPA. In time perception, the child shall identify which activity takes a long time or a short time, a choice between 2–3 pictures, with increasing difficulty. In time orientation, there are questions about days, the week, the month, or the year, and placing picture sequences in the correct temporal order. In objective time, it includes items to tell time from a clock and calculate time. Items in time management capture what activities one could manage to do, calculate, and allocate time to activities. KaTid-Youth contains the same categories, but consists of 59 items, 33 of which are the same as in KaTid-Child. KaTid-Child and KaTid-Youth have been shown to have good validity and reliability in children and adolescents, with and without disabilities, and are useful for measuring change [34,42,44]. The two versions of KaTid measure the same construct, TPA [6]. An earlier cluster analysis indicated that the patterns of TPA mainly follow the chronological age of children without disabilities, all clusters differing as regards the level of TPA [8]. The level of TPA seems to be a more valid overall base than the type of diagnosis for the planning of interventions in DTM (ibid). KaTid-Child has been validated and tested for internal consistency in children with and without disabilities, with Cronbach Alphas of 0.78–0.86 [2,7]. KaTid has been used with TD children, for children with ADHD, Autism, mild or moderate level ID, Cerebral Palsy, and dual diagnosis [2,9,34]. In this study, KaTid-Child or KaTid-Youth was used, depending on the age of the child. However, for three of the older children with ID, KaTid-Child was used because the KaTid-Youth version was considered too difficult. The certified and clinically experienced occupational therapists estimated that KaTid-Youth could be used for all TD children regardless of age.

In the structured questionnaire Time-Parent scale, the parents rate their children’s DTM: understanding concepts of time, managing time, and adapting to time demands [2,7]. There are two versions: children aged 5–10 years and adolescents aged 10–17 years. Both questionnaires consist of 12 statements rated on a Likert frequency/agreement scale with four agreement response options scored from never/I do not agree at all (1p) to always/I completely agree (4p). There is also an option: do not know (0p). The statements in the two versions capture the same ability but are set to different degrees of difficulty and are customised to the different age ranges. The Time-Parent scale has been validated and tested for internal consistency in children and adolescents with and without disabilities, with Cronbach’s alphas of 0.79–0.86. It has been found to be psychometrically sound and significantly correlated with TPA as measured by KaTid [2,7] and can measure change [34].

Using the Autonomy scale of the instrument “Children’s participation in school, students’ assessment forms” [17,18], the children rated their autonomy. This scale is an adapted short version of the Autonomous Functioning Checklist Self-determination scale [48], where the autonomy items are based on Sigafoos et al. [49]. There are two versions: for children aged 7–12 years and for adolescents aged 13–17 years. Children rate their autonomy in everyday activities or situations that they usually do or decide upon by themselves, based on 23 statements (children’s version) and 30 statements (adolescents’ version). The items are divided into sections: daily routines, interacting with people, recreational and leisure choices, community involvement, and personal style. In the adolescents’ version, there is an additional section: education and planning for my working life. It has a Likert response scale for frequency: I do not do it (1), sometimes (2), often (3), and always (4). The Autonomy scale has been validated and tested for internal consistency with a Cronbach Alpha of 0.82 for children aged 7–12 years and 0.93 for adolescents aged 13–17 years [17]. There is also a short version, Autonomy Short, for children who need an even shorter questionnaire. It is based on 14 items with the same four response alternatives as the other versions. All seven items capturing autonomy in daily routines from the original children’s version are included, as well as some items concerning choices and interacting with people. Autonomy Short has been shown to be psychometrically sound in construct validity [7], and it has been demonstrated that a version using the first 12 items can be used to measure self-rated autonomy equally as well as the longer versions [8]. The Autonomy scale has been used in studies for children of different ages and with and without disabilities. In Almqvist et al. [17], 472 children with disabilities participated using the Autonomy scale, including children with visual disability, motor disability, and children with multiple disabilities. Of these children, 84 also had an impairment in cognitive skills, probably ID. The Autonomy short was previously used and validated in children aged 6–10 years, (*n* = 82) with and without disabilities for construct validity (person separation index = 1.28), reliability (item reliability = 0.94) and internal consistency (Cronbach’s a = 0.65–0.86) [7] and children aged 6 to 11 years with dysfunctions such as ADHD, autism, or physical or intellectual disabilities (*n* = 118) [2]. The autonomy short version was also used for children with IDs aged 10–17 years (*n* = 61). In this sample, reliability was fair (a = 0.68) [44]. In the present study, all three versions were used.

### 2.4. Ethical Aspects

The ethical guidelines stipulated by the Helsinki Declaration [50] guided the research process. Ethical approval for this study was granted by the Regional Ethical Review Board in Linköping for the children with ADHD (D-nr. 2012/166-31), for the children with ID and the TD children (D-nr. 2005/032) and by the Regional Ethical Review Board in Uppsala for the youth with ID and the TD children (Dnr. 2009/241). Written informed consent was obtained from all parents and from the older children/adolescents in the study.

### 2.5. Data Analysis

All data analysis was performed using IBM SPSS Statistics version 26, selecting a significance level of 0.05 and a confidence interval of 95%.

#### 2.5.1. Comparisons Between Groups

To respond to research question 1, variable-based analysis was used to compare TPA, DTM, and self-rated autonomy between children with ADHD, ID, and TD children. To make it possible to compare the data for all children, some transformations of the different scales were needed [51]. Data collected with KaTid-Child (*n* = 37) were transformed into KaTid-Youth scores using a transformation scale, making the score sums comparable [6]. A calculation of the percentage of maximum scores was also made. Regarding the Time-Parent scale, there were two versions used. The sum of scores of the version of the Time-parent scale used for each child was transformed into a percentage of the maximum score. Three different versions of the autonomy scales were used. To compare autonomy for as many children as possible, the Autonomy Short and the version for 7–12-year-olds were combined into one (hereafter named “Autonomy-short”), including only those items that were identical in both versions. An analysis was conducted on the sum scores from the Autonomy-short version and the version for 13–17-year-olds, respectively, and on the percentage of maximum scores from both versions.

Group comparisons were made using one-way analysis of variance (ANOVA) with Tukey post-hoc tests for multiple comparisons [52]. A bootstrapping procedure with 1000 replicated Cls was conducted to correct for subgroup heterogeneity and small sample size. Reported *p*-values are adjusted accordingly [52]. Between-group analysis of Effect size (ES) for the different groups was calculated using Cohen’s *d*, with ES *d* = 0.2–0.5 representing a small effect, *d* = 0.5–0.8 medium, and *d* > 0.8 large effect [53].

#### 2.5.2. Cluster Analysis

To respond to research questions 2, 3, and 4, a hierarchical cluster analysis of patterns of TPA for all children was conducted using SLEIPNER 2.0 [54] with Ward’s method of agglomeration. Data used in the cluster analysis were scores from the four subcategories of KaTid-Child and KaTid-Youth. Since different versions of KaTid with different sum scores on each subcategory were used for the children, it was necessary to convert to a percentage of the maximal sum score for each subcategory in the two versions of KaTid. An argument for this is the fact that there is no transformation scale (as was used in the comparison analysis) for the subcategories. Also, it is the patterns of TPA being investigated in this second analysis rather than the actual difficulty of the subcategory. In this study, the following criteria were used to identify the number of clusters: (i) a homogeneity coefficient of at most 1.00 (preferably <0.50), (ii) a percent of the total explained error sums (ESS) of at least 67% and (iii) a distinctive break in the scree plot indicating a large increase in ESS and the number of clusters to retain before the increase and, (iiii) split sample replicability through a total rerun of the cluster analysis on a random half of the sample of at least 0.30, the mean in ASED between the clusters in the two solutions [54,55] using the Random module. Three random samples were run (*n* = 70), resulting in one cluster solution with five clusters and two solutions with four clusters. The first cluster solution included two residue cases, while the other two did not include any residue cases. After removal of the two residual cases in the first cluster solution, a reliability analysis was run on the random split samples, demonstrating an average pairwise matching between the clusters of 0.34. Eventually, a four-cluster solution was chosen, as it appeared to best match the data according to the above criteria. These statistical criteria provided rough guidance regarding the number of clusters to retain; however, a theoretical examination of the cluster solution guided the decision. The resulting four-cluster solution matched the theoretical underpinnings of the study and seemed meaningful in relation to earlier findings [2,8] of TPA of children with and without disabilities.

Comparisons were made concerning differences in age, TPA, DTM, and autonomy, and between clusters, using one-way analysis of variance (ANOVA) and bootstrapping (see above). Chi-square tests were used to analyse differences between the clusters regarding sex and diagnosis [56]. To determine whether there were differences within the clusters, subgroup analyses were also performed regarding age and sex in the two clusters with the largest numbers of participants.

## 3. Results

The mean age of the children with ADHD was 11.5 yr. (*SD* 1.93), children with ID 11.6 yr. (*SD* 1.83) and for the TD children 11.5 yr. (*SD* 1.90). Most of the children in each group were boys (*n* = 32, 68%).

### 3.1. Missing Data

There was no missing data in TPA and DTM. In self-rated autonomy, there was missing data from one child with ADHD and from nine children with ID. A sensitive analysis did not show any significant differences using the whole sample compared to only using data from children with no missing data.

### 3.2. Differences in TPA, DTM, and Autonomy Between Groups

#### 3.2.1. Time-Processing Ability

There were statistically significant differences between the groups, F (2138) = 84.49, *p* < 0.001. TD children had the highest scores for TPA, measured by KaTid and all data converted to KaTid-Youth, with a mean of 51.6 (*SD* 8.4). Children with ADHD had the second-highest scores for TPA (mean 42.4, *SD* 11.0), and children with ID had the lowest scores for TPA (mean 23.2, *SD* 12.6) (Table 1). There were statistically significant differences between children with ADHD and TD children (*p* < 0.001), children with ADHD and children with ID (*p* < 0.001), and children with ID and TD children (*p* < 0.001), with large ES on all comparisons (Table 1).

#### 3.2.2. Daily Time Management

Statistically significant differences in daily time management were found between the groups, F (2, 138) = 27.45, *p* < 0.001. TD children had the highest scores for DTM (mean 66.2, *SD* 13.8) according to the percent of maximum score of the Time-Parent scale (Table 2), children with ID had the second highest scores for DTM (mean 57.1, *SD* 17.1), and children with ADHD had the lowest scores for DTM (mean 44.6, *SD* 10.2). There were statistically significant differences between children with ADHD and TD children (*p* < 0.001), between children with ADHD and children with ID (*p* < 0.001 and between TD children and children with ID (*p* = 0.007). The ES were large or medium (Table 2).

#### 3.2.3. Autonomy

There were statistically significant differences between the groups in autonomy, F (2, 128) 30.58, *p* < 0.001. TD children had the highest scores in both autonomy versions, followed by children with ADHD (Table 3). Children with ID had the lowest scores in both autonomy scale versions. Using percent of maximum score based on both versions of autonomy, TD children had the highest scores (mean 80.7, *SD* 10.4), children with ADHD had the second highest scores (mean 76.0, *SD* 9.1), and children with ID had the lowest scores (61.3, *SD* 15.3). There were statistically significant differences between children with ADHD and children with ID (*p* < 0.001) and between TD children and children with ID (*p* < 0.001), with large ES.

### 3.3. Patterns of TPA in Children with ADHD, Children with ID, and the TD Children

The cluster analysis resulted in four clusters with different patterns of TPA, all based on the four subcategories of TPA. The evaluation of the explained error sums of squares (69.70%) demonstrated an acceptable cluster solution. The homogeneity coefficient of the groups differed between 0.451 (Cluster C), 0.706 (Cluster B), 0.709 (Cluster A), and 1.131 (Cluster D), which is acceptable (Table 2).

#### 3.3.1. Outliers

There were two residuals, i.e., children with values that exceeded the threshold value for pattern inclusion, found in both ID children. In total, 139 participants were included in the clusters.

#### 3.3.2. Descriptives

**Figure 1:**
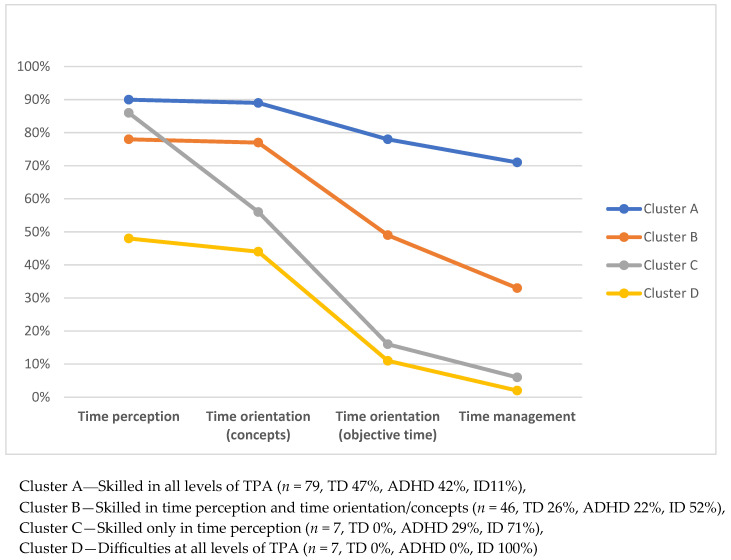
Time-processing ability (TPA) in percent of maximum scores in each subcategory of KaTid: Time perception, Time orientation (concepts), Time orientation (objective time), and Time management; in all four clusters.

There were statistically significant differences between clusters in age, F (3.135) = 3.25, *p* = 0.024. The distribution of sex in the clusters varies between 57% and 86% boys. Still, there were no statistically significant differences in sex between the clusters (*p* = 0.756). There were statistically significant differences between clusters according to diagnosis (*p* < 0.001). Children with ADHD were represented in three of the four clusters, and TD children in two. There were children with ID in all clusters. All clusters showed the highest proportion of correct answers at the lower levels of TPA, i.e., time perception and time orientation/concepts, and a reduced ability at the higher levels, i.e., time orientation/objective time and time management (Figure 1). There were statistically significant differences in TPA between clusters, F (3135) = 185.40, *p* < 0.001.

Descriptions of the participants in each cluster according to number, sex, diagnosis, mean age, TPA (total score, time perception, time orientation (concepts and objective time), and time management). DTM and self-rated autonomy are presented in Table 4. The results for each cluster are presented separately below.

##### Cluster A—(Skilled in All Levels of TPA)

This cluster is characterized by children skilled in all levels of TPA: time perception, time orientation (concepts and objective time), and time management (Figure 1). According to TPA, this cluster has a mean of 82% of total scores, which is the highest in the sample. Cluster A is the largest (*n* = 79) and contains children from all groups, but children with ID are underrepresented (11%). TD children make up the largest proportion (47%), followed by children with ADHD (42%). Mean age of the children is 11.8 (*SD* 1.92; range 8.72–15.73), i.e., the oldest children except for Cluster D (containing only children with ID). TD children in this cluster are the oldest of the TD children in the sample. Subgroup analysis of age in Cluster A shows that TD children have a mean age of 12.0 years, children with ADHD 11.7 years, and children with ID 11.5 years.

##### Cluster B—(Skilled in Time Perception and Time Orientation/Concepts)

This cluster is characterized by children skilled in time perception and time orientation/concepts, but who have difficulties with time orientation/objective time and time management (Figure 1). According to TPA, this cluster has a mean score that is the second-highest, 58% of total scores. Cluster B is the second largest (*n* = 46) and contains children from all groups, children with ID being overrepresented (52%). The distribution of TD children and children with ADHD is relatively even (26% and 22%, respectively). The mean age of the children is 10.9 (*SD* 1.66; range 8.62–15.14), which means that it is the cluster with the youngest children. The TD children in this cluster are the youngest of the TD children in the sample. Subgroup analysis of age in Cluster B shows a mean age for TD children of 9.8, children with ADHD of 11.2, and children with ID of 11.2.

##### Cluster C—(Skilled Only in Time Perception)

This cluster is characterized by children skilled only in time perception and who have considerable difficulties with time orientation (both concepts and objective time) and time management (Figure 1). According to TPA, this cluster has a mean of 39% of total scores, i.e., the second lowest in the sample. Cluster C is small (*n* = 7) and contains children with ADHD and children with ID, with children with ID being overrepresented (71%). Mean age is 11.1 (*SD* 1.42; range 9.44–13.50). Children with ID are older (*M* = 11.6) compared to children with ADHD (*M* = 9.7).

##### Cluster D—(Difficulties at All Levels of TPA)

This cluster is characterized by children having difficulties at all levels of TPA, but mostly with time orientation/objective time and time management (Figure 1). According to TPA, this cluster has a mean of 24% of total scores, which is the lowest in the sample. Cluster D is small (*n* = 7) and contains only children with ID. Children in this cluster are the oldest in the sample, with a mean age of 12.5 (*SD* 2.24; range 9.66–15.58).

#### 3.3.3. Diagnostic Category and Age Related to Pattern of TPA

Children with ADHD are represented in three clusters, i.e., not in cluster D (Difficulties at all levels of TPA). Most children with ADHD are found in Cluster A (Skilled in all levels of TPA), in which they are about as numerous as the TD children, and in Cluster B (Skilled in time perception and time orientation/concepts).

Children with ID are represented in all clusters, with the largest numbers found in Cluster B (Skilled in time perception and in time orientation/concepts), where they are overrepresented. They are also overrepresented in Cluster C, containing children skilled in time perception but having difficulties with all other levels of TPA, and in Cluster D (Difficulties at all levels of TPA), containing children with the lowest scores of TPA.

The TD children are represented in two clusters: Cluster A (Skilled in all levels of TPA) (79% of TD children) and Cluster B (Skilled in time perception and time orientation/concepts).

Children in Cluster B (Skilled in time perception and time orientation/concepts) have the lowest mean age (10.9), followed by Cluster C (Skilled only in time perception) (11.1) and Cluster A (Skilled in all levels of TPA) (11.8). Children in Cluster D (Difficulties at all levels of TPA) have the highest mean age (12.5). The age range differs between clusters; Cluster A has the largest range (8.72–15.73) and Cluster C the smallest (9.44–13.50).

#### 3.3.4. Daily Time Management and Autonomy

DTM, as rated by the parents, is almost the same in all clusters, the exception being children in Cluster D (Difficulties at all levels of TPA), who have the lowest score (40% of the maximum score). There were no statistically significant differences between the clusters.

There were significant differences in autonomy, as rated by the children themselves, between the clusters, F (3125) = 15.98, *p* < 0.001. Children in Cluster A (Skilled in all levels of TPA) have the highest autonomy scores (78% of maximum score), followed by children in Cluster B (Skilled in time perception and time orientation/concepts) (71%), Cluster C (Skilled only in time perception) (60%) and the lowest scores in Cluster D (Difficulties at all levels of TPA), with 42% of maximum score. There are statistically significant differences between Cluster A and B (*p* = 0.003), Cluster A and C (*p* = 0.001), Cluster A and D (*p* < 0.001), and Cluster B and D (*p* < 0.001).

## 4. Discussion

The aim of this study was to investigate daily time management (DTM), Time processing ability (TPA), and self-rated autonomy in everyday activities in children aged 9–15 years with ADHD, compared to children with ID and to TD children. The results will be discussed in relation to the research questions.

### 4.1. Differences in TPA, DTM, and Self-Rated Autonomy in Groups Based on Diagnosis

Children with ADHD had statistically significantly lower scores on TPA and DTM compared to TD children. Parents of children with ADHD rated their children’s DTM as low, even lower than that of children with ID. These results indicate that children with ADHD may have both deficits in cognitive functioning regarding TPA and problems in daily activities related to time. The problems in TPA are in line with experimental studies showing deficits in time perception [24,26,27,28,57], temporal foresight [28], and time-based prospective memory tasks [30]. This study adds that the children have problems not only in time perception but also in other categories of TPA. Further, this study adds that the children have problems specifically in DTM and are possibly more outspoken than children with ID. Other studies have shown problems in organizing and planning school activities [31,58], daily activities at home [34,35], and overall adaptive functioning [32] among children with ADHD. It is likely that these problems are related to, and in the latter two, possibly due to the low DTM. One explanation of the very low parent-rated DTM in children with ADHD may be that the parents may have expectations and demands comparable to TD children, while parents of children with ID more often adapt their expectations to the child’s abilities. Given that children with ID had the lowest TPA in the sample, we might expect the parents of children with ID to rate their children’s DTM as lowest, but they did not. One explanation could be that parents of children with ID have low expectations of their child’s DTM because of their known limitations in intellectual and adaptive functioning, the criteria for the diagnosis [37]. In addition, it is clinically known that children with ID often have difficulties related to time [38]. In Scandinavia, persons with ID are the target group who have had access to time-assistive devices for the longest, especially low-tech products such as adapted weekly schedules and/or activity schedules [42,43,59,60]. So, another potential explanation may be that many of the children with ID in this sample had access to time-assistive products to compensate for deficits in TPA and to support their DTM, compared to only three children with ADHD. The usefulness and effect of time-assistive products on DTM have been demonstrated in several studies [34,35,41,42,43], supporting this explanation

Children with ADHD had a lower mean score for autonomy than TD children. Janeslätt et al. [8] showed in a study of children aged 5–10 years, with and without a diagnosis, that a child’s self-rated autonomy was related to their TPA. The higher the TPA a child had, the higher they self-rated their autonomy. Peny-Dahlstrand et al. [14] also found that high observed executive functions were related to high levels of self-rated autonomy in children with spina bifida. That both decisional and executive autonomy are dependent on cognitive functions has been found in several studies [12,13,14]. The fact that children with ID in the present study, who had the lowest scores for TPA, also had the lowest scores for self-rated autonomy agrees with this. The lower levels of TPA in children with ADHD and ID in our study, compared to TD children, could therefore be an explanation for the lower scores on self-rated autonomy compared to the TD children. The fact that autonomy is suggested to be a prerequisite for participation [12,16] highlights the importance of interventions supporting cognitive functions, including TPA, in children with ADHD and ID to enhance their autonomy and thereby support their participation.

### 4.2. Patterns of TPA in Children with and Without Diagnosis

However, the differences between diagnosis groups described above do not consider the diversity within the children with the same diagnosis. Therefore, we went further with a person-oriented approach, based on all four categories of time-processing ability in children with and without a diagnosis, and it revealed four different clusters of TPA. All the clusters showed a similar overall pattern, with the highest scores being for time perception, and a decreased ability one by one for the subsequent higher levels of TPA. Investigating such patterns of categories, rather than general (diagnosis-based) groups that vary widely in TPA, could give important knowledge to plan and implement individualised efforts to strengthen TPAs. Our findings are in line with previous studies showing a development of TPA for TD children, starting with perception of time, followed by time orientation, and later time management [3,7]. The same pattern was found in a study including younger children, aged 5–10 years, with and without disabilities [8]. This study adds that the patterns of TPA mainly follow the difficulty of each category of TPA, also in older children with a variety of capacities in TPA. This is in line with earlier research, supporting earlier findings that TPA can be seen as one construct with categories forming different levels of time-processing [2,6].

An interesting finding was that in the present study, all clusters showed a distinct difference in the decrease between time orientation/concepts and time orientation/objective time. This difference was more evident in the clusters with lower overall levels of TPA. This indicates that there are subcategories within time orientation; that time concepts are easier to manage than understanding what objective time is. This is in line with Janeslätt et al. [8], who raised the issue of time orientation as two different strands. Based on this, we suggest further research with larger samples.

### 4.3. Specific Patterns of TPA

In the present study, there were statistically significant differences between clusters concerning diagnosis. Nevertheless, there are no signs of differences in the overall pattern of TPA, indicating that children with ADHD and ID have the same pattern of TPA development compared to TD children, but for some of these children at a slower pace. The same was found in Janeslätt et al. [8], where children with disabilities were older than TD children in the same cluster. This indicates a need for targeted and early intervention to enhance the development of TPA (ibid). Training of organizational, time management, and planning skills (OTMP) is a well-established treatment in children and adolescents with ADHD [61], and interventions focusing on TPA, as described in Wennberg et al. [34], as a complement, are therefore needed. Otherwise, the functional impact (of having ADHD or ID) may increase with age [62].

Children with ADHD were represented in three of the four clusters. It is worth noticing that none of the three clusters A, B, and C that included children with ADHD had pronounced difficulties in time perception. This contrasts with studies showing that time perception, including time sense, in children with ADHD differs compared to typically developing children [23,24] and that their ability to discriminate and reproduce time intervals and to make retrospective time estimations was reported as impaired [25,26,27]. First, it is essential to note that all children with ADHD in this study were on a steady ADHD medication. Abikoff et al. [63] could show that ADHD medication (MPH-OROS) significantly reduced the children’s deficits in organizational, time management, and planning behaviors, and with significant reductions in core ADHD symptoms. Still, most of the children (61%) continued to show significant impairments in organization, time management, and planning. Thus, the children in our study could have reductions in ADHD symptoms, including problems with time sense, due to medication, but still have problems enough to be included in the RCT study. The fact that in clusters A and B, the proportion of children with ADHD is roughly the same as the proportion of children with TD may possibly offset the difficulties experienced by children with ADHD. Also, the items in KaTid measuring time perception include two items measuring time sense and five items measuring the subjective experience of durations of activities. Possibly knowing the duration of familiar activities is less problematic and could even be helpful for children with ADHD, even if time sense is lacking? Anyhow, the overall results further emphasize the diversity of TPA among children with ADHD. This contrasts with the TD children, who, not surprisingly, were only represented in the clusters with the most able children. The fact that children with ADHD were more than a year older than TD children in Cluster B (Skilled in time perception and time orientation/concepts) and the presence of children with ADHD in Cluster C (Skilled in only time perception) indicates a delayed TPA for some children with ADHD, and some aspects of TPA, compared to TD children. This is in accordance with studies showing a delay in maturation in the prefrontal cortical regions important for executive functions, including time management, in children with ADHD [21,22].

Cluster C, Skilled only in time perception, draws attention as the pattern seems to differ from the others. The discrepancy in time orientation/concepts is so much lower than could be expected. Still, the order of the categories is the same as in clusters A, B, and D. Children with ID are present in all four clusters, but overrepresented in Cluster C. They were also, like the children with ADHD, more than a year older than TD children in the Cluster B (Skilled in time perception and time orientation/concepts). Cluster D, which contains the oldest children in the sample and where the children have the lowest TPA, contains only children with ID. Children in this cluster probably have a disability that affects most situations in everyday life, at home, at school, and during leisure time. Still, it is noteworthy that some children with ID are found in Cluster A (Skilled in all aspects of TPA). Taken together, children with ID in this study show a great variety in TPA, not explained by age, indicating a need for individual measurement and tailored interventions for children with ID. Further, the results indicate that time-assistive products are useful to support DTM in these children. Possibly adding training of low levels of TPA, as described by Janeslätt and colleagues [44], would also benefit some of these children.

### 4.4. Patterns of DTM and Self-Rated Autonomy

Except for Cluster D (Difficulties at all levels of TPA), there were almost no differences in parent-rated DTM between clusters, although the clusters differed in TPA. This result conflicts with a previous study showing that DTM is related to a child’s TPA [8]; however, these children were younger (aged 5- 10 years), and the diversity of children’s diagnoses was larger including children with autism spectrum disorder (*n* = 14), Spina Bifida and Cerebral Palsy (*n* = 20), mild or moderate level ID (*n* = 20), children with ADHD (*n* = 13) and TD children (*n* = 89). These differences between the present study and Janeslätt et al. [8] are probably the reason for the disparity between the results. As TPA is the cognitive functioning and DTM is the daily functioning in the aspects of managing time, there could also be other explanations. According to Janeslätt et al. [2], parents’ ratings of their children’s DTM explained 25% of the variation in TPA. Other explanations could be, for example, environmental factors, including support for understanding time provided by time-assistive devices, or support from parents and teachers [2,4,8].

Interestingly, in the present study, there are contradictory results concerning DTM. Group analysis of differences between TD children, children with ADHD and children with ID resulted in statistically significant differences in DTM, but this cannot be seen in the comparisons of clusters. This leads to a need for further research on TPA and DTM in children with ADHD and ID, with larger samples.

In contrast to DTM, there were differences in the clusters concerning self-rated autonomy. The patterns of autonomy seemed to follow the level of TPA. Children in clusters with high scores of TPA also have higher scores of autonomy. This is in line with the results in Janeslätt et al. [8] concerning children aged 5–10 years with and without a diagnosis.

## 5. Methodological Issues/Limitations

A strength of the study is that two different methods were used for analysing the data: a group comparison [46] to answer the broader question of TPA in children with functional variations compared to TD children, and a person-oriented approach [55] to investigate dimensional aspects of TPA. Comparing two or more groups on a specific subject is a useful method for revealing group differences [46]. According to Bergman et al. [55], a person-oriented approach gives a thorough description of individual differences and enables a translation of the results into properties characterising individuals. This gives a possibility to plan and offer individualized interventions based on the individual’s difficulties and not only based on diagnosis.

In the present study, both parent ratings and self-ratings were used. This gives a broad picture of children’s functioning in everyday life. Obtaining information from both children and their parents provides opportunities for both perspectives and could give important information concerning the child’s health and well-being [64,65].

The recruitment of children with ADHD had the purpose of finding children who could benefit from the time-related intervention described in Wennberg et al. [34]. As a result, the children’s difficulties with time in the current study cannot fully be generalized to all children with ADHD.

A limitation of this study is that the specific cognitive level of children with ID was not known, as the ethical application for children with ID included diagnoses, but not specific IQ. However, since KaTid assessment could be performed on all children with ID, severe or profound ID could be excluded. There is also a lack of sociodemographic data for children with ID, which may restrict further group comparisons between children with ADHD and children with ID.

A limitation is also the number of dropouts of self-ratings of autonomy in children with ID. We do not know the reasons, except for two children (who did not understand the questions and did not want to answer). One explanation of the dropouts could be that the number of statements was too time-consuming and demanding for the children since this self-rating of autonomy was often performed at the same time as the KaTid-measurement, and self-rating of DTM (not included in the present study). Another explanation could be that the children with ID were unfamiliar with self-reporting questionnaires. Still, the self-rating of autonomy contributes important and complementary information.

Another limitation is that two of the four clusters had few participants, which was the outcome of the cluster analysis performed. This could call for a more cautious interpretation of the results from these clusters. However, the primary aim of the cluster analyses was not to compare, but rather to describe the differing patterns of each cluster. The low number of participants in clusters C and D may indicate that these patterns are less common, and these profiles should be interpreted as patterns of co-occurring characteristics rather than homogenous or generalizable subgroups. This could be investigated in further research.

## 6. Conclusions

- This study deepens and expands the knowledge of time deficits in children and adolescents with ADHD and ID in relation to the whole construct of time-processing ability, including time perception, time orientation, and time management.
- Children with ADHD, as a group, have lower TPA and autonomy compared to typically developing children, but in daily time management, they are also lower than children with ID.
- The present study shows that children with ADHD and ID in this sample have the same overall pattern of TPA but have a lower TPA in relation to the typically developing children. This discrepancy could affect their DTM and autonomy.
- The overall findings indicate a need to measure children’s ability in each category of TPA and the DTM, to tailor a suitable intervention for each child.

## Figures and Tables

**Table 1:** Group differences for time-processing ability (TPA) as measured with KaTid, mean (M), (SD), 95% Confidence interval (CI), numbers of participants in each group (*n*), *p*-values, effect size, in typically developing children (TD), children with ADHD and children with ID (*n* = 47 per group), all data converted to KaTid-Youth version.

	TD	ADHD	ID	*p*-Values	Effect Size
KaTid^®^ (maximum score 65) M (SD) CI/*n*	51.6 (8.4) 48.87–53.99/47	42,4 (11.0) 39.26–45.71/47	23.2 (12.6) 19.45–26.86/47	*p* < 0.01 ^a; b; c^	0.92 ^a^; 1.63 ^b^; 2.7 ^c^
Percentage of maximum score M (SD) CI/*n*	79.3 (13.0) 75.12–83.37/47	65.2 (17.1) 60.70–69.64/47	36.5 (19.5) 31.04–41.88/47	*p* < 0.01 ^a; b; c^	0.94 ^a^; 1.57 ^b^; 2.63 ^c^

Group differences in scores and percentage of maximum scores, ^a^ = TD children compared to children with ADHD, ^b^ = children with ADHD compared to Children with ID, ^c^ = TD children compared to children with ID. Effect size using Cohen’s d.

**Table 2:** Group differences for daily time management (DTM) measured with the Time-Parent scale, mean (M), (SD), 95% Confidence interval (CI), numbers of participants in each group (*n*), *p*-values, effect size, in typically developing children (TD), children with ADHD, and children with ID.

	TD	ADHD	ID	*p*-Values	Effect Size
Time-Parents scale 5–10 years version (maximum score = 44) M (SD) CI/*n*			30.0 (7.2) 26.64–33.65/20		
Time-Parents scale 10–17 years version (maximum score = 48) M (SD) CL/*n*	31.7 (6.6) 29.69–33.75/47	21.4 (4.9) 20.09–22.72/47	23.4 (6.4) 21.24–25.78/27	*p* < 0.01 ^a; c^; NS ^b^	1.79 ^a^; 0.35 ^b^; 1.28 ^c^
Percentage of maximum score (based on 5–10 years version and 10–17 years version respectively) M (SD) CL/*n*	66.2 (13.8) 62.32–70.13/47	44.6 (10.2) 41.69–47.71/47	57.1 (17.5) 52.48–61.88/47	*p* < 0.01 ^a; b^; *p* < 0.05 ^c^	1.8 ^a^; 0.90 ^b^; 0.58 ^c^

Group differences in scores and percentage of maximum scores, ^a^ = TD children compared to children with ADHD, ^b^ = children with ADHD compared to Children with ID, ^c^ = TD children compared to children with ID. Effect size using Cohen’s d.

**Table 3:** Group differences for self-rated autonomy measured by Autonomy scale, mean (M), (SD), 95% Confidence interval (CI), numbers of participants in each group (*n*), *p*-values, effect size, in typically developing children (TD), children with ADHD, and children with ID.

	TD	ADHD	ID	*p*-Values	Effect Size
Autonomy scale 5–12 years version and Short version (maximum score = 48) M (SD) CI/*n*	39.1 (5.3) 36.83–40.80/34	36.4 (4.4) 34.86–38.00/33	30.6 (7.6) 27.76–33.39/29	*NS* ^a^; <0.05 ^b^; <0.01 ^c^	0.56 ^a^; 0.97 ^b^; 1.32 ^c^
Autonomy scale 13–17 years version (maximum score = 112) M (SD) CI/*n*	94.5 (9.9) 89.06–99.84/13	91.2 (11.6) 84.82–97.46/13	64.1 (12.7) 56.26–72.07/9	*NS* ^a^, <0.01 ^b; c^	0.31 ^a^; 2.23 ^b^; 2.69 ^c^
Percentage of maximum score (based on 5–12 years Short version and 13–17 years version, respectively) M (SD) CI/*n*	80.7 (10.4) 77.17–83.61/47	76.0 (9.1) 73.47–78.42/46	61.3 (15.3) 56.89–65.93/38	*NS* ^a^ <0.01 ^b; c^	0.48 ^a^; 1.20 ^b^; 1.51 ^c^

Group differences in scores and percentage of maximum scores, ^a^ = TD children compared to children with ADHD, ^b^ = children with ADHD compared to Children with ID, ^c^ = TD children compared to children with ID. Effect size using Cohen’s d.

**Table 4:** Demographics of children in the clusters *.

	Cluster A	Cluster B	Cluster C	Cluster D	All Clusters
Homogeneity	0.709	0.706	0.451	1.131	
*n* (% of the sample)	79 (56.8)	46 (33.2)	7 (5)	7 (5)	139 (100)
Sex *n* (% in each cluster)					
Girls	25 (57)	15 (34)	1 (2)	3 (7)	44 (100)
Boys	54 (57	31 (33)	6 (6)	4 (4)	95 (100)
Diagnosis/no diagnosis *n* (%)					
TD	37 (78.7)	10 (21.3)			47 (100)
ADHD	33 (70.2)	12 (25.5)	2 (4.3)		47 (100)
ID	9 (20.0)	24 (53.3)	5 (11.1)	7 (15.6)	45 (100)
Age *M* (SD; min-max)					
All children in the cluster	11.8 (1.92; 8.72–15.73)	10.9 (1.66; 8.62–15.14)	11.1 (1.42; 9.44–13.50)	12.5 (2.24; 9.66–15.58)	11.5 (1.88; 8.62–15.73)
TD	12.0 (1.86; 9.00–15.64)	9.8 (0.73; 9.11–11.32)	-	-	
ADHD	11.7 (1.99; 8.72–15.73)	11.2 (1.79; 8.62–15.14)	9.7 (0.30; 9.44–9.86)	-	
ID	11.5 (2.07; 9.17–15.08)	11.2 (1.74; 8.66–14.92)	11.6 (1.28; 10.42–13.50)	12.5 (2.25; 9.66–15.58)	
TPA ^a^ *M* (SD)/*n*					
TPA Total score,	82 (8.42) /79	58 (8.85) /46	39 (4.15)/7	24 (8.71)/7	
Time perception	88 (8.26)	78 (9.31)	86 (8.96)	48 (13.32)	
Time orientation/ concepts	89 (8.66)	76 (9.08)	56 (6.64)	44 (14.53)	
Time orientation/ objective time	78 (13.87)	48 (14.54)	16 (10.38)	11 (13.78)	
Time management	71 (17.88)	32 (13.43)	6 (8.21)	2 (3.41)	
DTM ^b^ *M* (*SD*)	57 (16.3) /79	56 (16.7)/46	60 (14.2)/7	40 (14.4)/7	
Autonomy ^c^ *M* (SD)	78 (10.7) /74	71 (12.6)/43	60 (16.1)/7	42 (9.1)/3	

* Two ID cases were excluded as residuals. Time-processing ability (TPA) ^a^ as a percentage of total score and subscale scores, Daily time management (DTM) ^b^ as a percentage of total score, and self-rated Autonomy ^c^ as a percentage of total score, in the clusters.

## Data Availability

The data that support the findings of this study are available from Linköping University, but restrictions apply to the availability of these data, which were used under license for the current study. Data are thus not publicly available. Data are, however, available from the authors upon reasonable request and with permission of Linköping University.
